# A simple statistical model for prediction of acute coronary syndrome in chest pain patients in the emergency department

**DOI:** 10.1186/1472-6947-6-28

**Published:** 2006-07-06

**Authors:** Jonas Björk, Jakob L Forberg, Mattias Ohlsson, Lars Edenbrandt, Hans Öhlin, Ulf Ekelund

**Affiliations:** 1 Competence Center for Clinical Research, Lund University Hospital, Lund, Sweden; 2 Department of Clinical Sciences, Section for Emergency Medicine, Lund University Hospital, Lund, Sweden; 3 Department of Theoretical Physics, Lund University, Lund, Sweden; 4 Department of Clinical Physiology, Malmö University Hospital, Malmö, Sweden; 5 Department of Clinical Physiology, Sahlgrenska University Hospital, Gothenburg, Sweden; 6 Department of Cardiology, Lund University Hospital, Lund, Sweden

## Abstract

**Background:**

Several models for prediction of acute coronary syndrome (ACS) among chest pain patients in the emergency department (ED) have been presented, but many models predict only the likelihood of acute myocardial infarction, or include a large number of variables, which make them less than optimal for implementation at a busy ED. We report here a simple statistical model for ACS prediction that could be used in routine care at a busy ED.

**Methods:**

Multivariable analysis and logistic regression were used on data from 634 ED visits for chest pain. Only data immediately available at patient presentation were used. To make ACS prediction stable and the model useful for personnel inexperienced in electrocardiogram (ECG) reading, simple ECG data suitable for computerized reading were included.

**Results:**

Besides ECG, eight variables were found to be important for ACS prediction, and included in the model: age, chest discomfort at presentation, symptom duration and previous hypertension, angina pectoris, AMI, congestive heart failure or PCI/CABG. At an ACS prevalence of 21% and a set sensitivity of 95%, the negative predictive value of the model was 96%.

**Conclusion:**

The present prediction model, combined with the clinical judgment of ED personnel, could be useful for the early discharge of chest pain patients in populations with a low prevalence of ACS.

## Background

Unstable angina pectoris and acute myocardial infarction (AMI), together denoted acute coronary syndrome (ACS), are consequences of acute coronary artery disease with myocardial ischemia. Despite considerable progress in the treatment of ACS with antithrombotic drugs and catheter-based interventions (balloon angioplasty), the ability to diagnose ACS in the emergency department (ED) remains relatively poor. Since missed cases of ACS carry a high morbidity and mortality from heart failure and arrhythmia, the number of "rule-out" admissions are high, and some 7 or more out of 10 patients admitted with the suspicion of ACS do no not have it [1,2]. This large overadmission implies a unsatisfactory quality of care for the patients and a high cost for the health care system [3,4]. To improve the situation, new diagnostic methods such as immediate stress tests [5], myocardial perfusion imaging [6], echocardiography [7], and new blood tests have been suggested. In addition, decision support tools in the form of prediction models have been developed to help the physician handle the clinical information, and thereby to better triage and treat the patient. A large number of such models have been presented [8-24], and most of them have been focused on the detection of AMI. With the current ACS paradigm, however, models that predict the probability of AMI are less useful in routine ED care, where the likelihood of ACS (rather than AMI) is normally decisive for admission or discharge, and for immediate treatment. Also, many of these models need substantial input from the ED personnel [8-12,18], and hence are not ideally suited for implementation in standard care at a busy ED. Only one previous model, the ACI-TIPI [14], has been both easy to use and predictive of ACS.

The aim of this study was to develop a simple statistical model, based on ECG and clinical data available immediately at presentation, which predicts the likelihood for ACS among chest pain patients in the ED. The intention was to include ECG data in the form of simple amplitude measurements to allow for machine reading. A prediction model of this type could form the basis for development of a user-friendly decision support system for ED personnel.

## Methods

### Setting and patient material

Lund University Hospital, Sweden, is a 1200 bed institution which serves as the primary hospital for some 250,000 inhabitants, has a cardiac intensive care unit with 19 beds and an intermediate care ward with ECG monitoring at 19 beds. Percutaneous coronary intervention (PCI) and coronary bypass surgery (CABG) are available 24 hours/day. There is a traditional ED with approximately 50000 patients per year. During the patient inclusion period, there was no systematic diagnostic protocol for patients with suspected ACS, and no dedicated chest pain unit.

Six hundred and sixty-five consecutive visits for chest pain for which electronic ECG data could be retrieved were retrospectively included at the ED of Lund University Hospital from July 1 to November 20, 1997. Characteristics of the 634 unique patients are presented in table 1. From the clinical data collected for each patient, 18 variables available immediately at ED presentation (table 1) were chosen for further study based on their likely importance for ACS prediction.

**Table 1:** Characteristics of patients who come to the emergency department with acute chest pain, due to acute coronary syndrome (ACS; n = 130) or other causes (n = 504).

	ACS n (%)	Other causes n (%)	OR	95% CI
Age				
≥ 80 years	35 (26.9)	86 (17.1)	10	2.9 – 34
70 – 79 years	39 (30.0)	103 (20.4)	9.3	2.8 – 31
60 – 69 years	26 (20.0)	98 (19.4)	6.5	1.9 – 22
50 – 59 years	22 (16.9)	89 (17.7)	6.1	1.8 – 21
40 – 49 years	5 (3.8)	54 (10.7)	2.3	0.52 – 10
< 40 years	3 (2.3)	74 (14.7)	1.0	-
Male	83 (63.8)	279 (55.4)	1.4	0.96 – 2.1
Smoking status				
Current	29 (22.3)	98 (19.4)	1.3	0.78 – 2.3
Former	50 (38.5)	155 (30.8)	1.5	0.91 – 2.3
Unknown	12 (9.2)	74 (14.7)	0.74	0.36 – 1.5
Non-smoker	39 (30.0)	177 (35.1)	1.0	-
Hypertension				
Yes	47 (36.2)	114 (22.6)	2.0	1.3 – 3.0
Unknown	8 (6.2)	27 (5.4)	1.4	0.63 – 3.3
No	75 (57.7)	363 (72.0)	1.0	-
Diabetes	19 (14.6)	57 (11.3)	1.3	0.77 – 2.4
Angina pectoris^a^				
Yes, ≤ 1 month	4 (3.1)	5 (1.0)	3.8	1.0 – 15
Yes, > 1 month	56 (43.8)	174 (34.5)	1.5	1.0 – 2.3
No	68 (53.1)	325 (64.5)	1.0	-
Congestive heart failure	20 (15.4)	79 (15.7)	0.98	0.57 – 1.7
Previous myocardial infarction				
Yes, ≤ 6 months	13 (10.0)	19 (3.8)	3.2	1.5 – 6.8
Yes, > 6 months	37 (28.5)	107 (21.2)	1.6	1.0 – 2.6
No	80 (61.5)	378 (75.0)	1.0	-
Previous PCI	4 (3.1)	21 (4.2)	0.73	0.25 – 2.2
Previous CABG	10 (7.7)	55 (10.9)	0.68	0.34 – 1.4
Treated with cardiac drugs^b^	85 (65.4)	282 (56.0)	1.5	0.99 – 2.2
Chest discomfort at presentation	85 (65.4)	238 (47.2)	2.1	1.4 – 3.2
Symptom duration^c^				
0 – 6 h	100 (76.9)	261 (52.0)	5.4	2.7 – 11
7 – 12 h	16 (12.3)	59 (11.8)	3.8	1.6 – 8.8
13 – 24 h	4 (3.1)	42 (8.4)	1.3	0.40 – 4.5
> 24 h	10 (7.7)	140 (27.9)	1.0	-
Tachypnea	13 (10.0)	27 (5.4)	2.0	0.98 – 3.9
Lung rales	12 (9.2)	23 (4.6)	2.1	1.0 – 4.4
Systolic bp < 100 mmHg^d^	4 (3.1)	4 (0.8)	3.9	0.97 – 16
Diastolic bp < 70 mmHg^e^	16 (12.9)	36 (7.3)	2.0	1.0 – 3.7
Heart rate^f^				
> 120 bpm	4 (3.1)	12 (2.4)	1.3	0.41 – 4.1
< 50 bpm	3 (2.3)	10 (2.0)	1.2	0.32 – 4.3
50 – 120 bpm	123 (94.6)	480 (95.6)	1.0	-

^a ^Data on angina pectoris were missing for two patients with ACS.

^b ^ACE-inhibitors, ASA, Calcium antagonists, Betablockers, Long-acting Nitrates or Diuretics.

^c ^Data on symptom duration were missing for two patients without ACS.

^d ^Data on systolic blood pressure were missing for 8 patients (one with and 7 without ACS).

^e ^Data on diastolic blood pressure were missing for 15 patients (6 with and 9 without ACS).

^f ^Data on heart rate were missing for 2 patients without ACS.

### Reference standard

The ED visit ended in hospitalization in 422 (67%) cases, and among these a discharge diagnosis of ACS was assigned in only 130 (31%), of which 28 (22%) had Q-wave AMI, 53 (41%) had non-Q-wave AMI and 49 (38%) unstable angina. Discharge diagnoses were made by the senior ward physician or the ED physician (in cases discharged from the ED), reviewed by a senior research nurse, and when ambiguous, further reviewed by a senior cardiologist (HÖ). In the review of diagnoses for cases discharged from the ED, available data from the patient records indicated that the rate of missed diagnosis of ACS was low (not more than 2%). AMI was defined by the WHO criteria [25] where the biochemical criterion was at least one measurement of CK-MB>10 μg/l or Troponin T>0.1 μg/l. The criteria for unstable angina were ischemic symptoms (chest pain >15 min., syncope, acute heart failure or pulmonary edema) together with at least one of the following: a) Electrocardiogram (ECG) changes: transient or persisting ST segment depression (≥ 1 mm) and/or T-wave inversion (≥ 1 mm) without developing Q waves or loss of R wave height, or b) Biochemical markers: CK-MB 5–10 μg/l or Troponin T 0.05–0.1 μg/l.

This study was approved by the Lund University Research Ethics Committee.

### Electrocardiogram

Two methods of ECG analysis were used, machine reading and expert reading.

#### Machine reading

The 12-lead ECGs were recorded by the use of computerized ECG recorders (Siemens-Elema AB, Solna, Sweden). The ECG measurements were obtained from the measurement program of the ECG recorders. From the leads I, aVF and V2, the two measurements ST-J amplitude and ST amplitude 3/8 were selected for further analysis. These measurements were selected to detect ST-elevation patterns caused by ischemia in the anterior, septal, inferior or lateral wall. The ST amplitude 3/8 was obtained by dividing the interval between ST-J point and the end of the T wave into eight parts of equal duration. The amplitudes at the end of the third interval were denoted ST amplitude 3/8. ECG data were entered in the logistic regression models as categorical variables, with the grouping defined in Table 2. The categorisation was based on widely used ECG criteria for ST-elevation and depression [26].

**Table 2:** Characteristics of the electrocardiogram for patients who come to the emergency department with acute chest pain, due to acute coronary syndrome (ACS; n = 130) or other causes (n = 504). I-STamp, ST amplitude in lead I; aVF-STamp, ST amplitude in lead aVF; V2-STamp, ST amplitude in lead V2; STamp38, ST amplitude at the end of the third out of eight equal intervals between the ST-J point and the end of the T wave.

	ACS n (%)	Other causes n (%)	OR	95% CI
I-Stamp				
I-STamp > 50 and I-STamp38 > I-STamp	7 (5.4)	12 (2.4)	2.4	0.94 – 6.3
I-STamp < -100 and I-STamp38 < I-STamp	7 (5.4)	6 (1.2)	4.9	1.6 – 15
None of above	116 (89.2)	486 (96.4)	1.0	-
aVF-STamp^a^				
aVF-STamp > 100 and aVF-STamp38 > aVF-STamp	13 (10.1)	4 (0.8)	14	4.6 – 45
aVF-STamp < -100 and aVF-STamp38 < aVF-STamp	4 (3.1)	3 (0.6)	5.9	1.3 – 27
None of above	112 (86.8)	497 (98.6)	1.0	-
V2-STamp^b^				
V2-STamp > 200 and V2-STamp38 > V2-STamp	17 (13.2)	27 (5.4)	3.0	1.5 – 5.7
100 < V2-STamp ≤ 200 and V2-STamp38 > V2-STamp	26 (20.2)	95 (18.9)	1.3	0.78 – 2.1
V2-STamp < -100 and V2-STamp38 < V2-STamp	6 (4.7)	5 (1.0)	5.6	1.7 – 19
None of above	80 (62.0)	376 (74.8)	1.0	-

^a ^One patient with ACS had missing value on aVF-STamp.

^b ^Two patients (one with and one without ACS) had missing value on V2-STamp.

#### Expert reading

For comparison, ECG for 628 of the 634 unique patients were retrieved at a later point in time, and 608 of these ECGs were possible to assess. Two physician experts first classified the 608 ECGs, which originated from 120 patients with ACS and 488 patients without ACS, independently of each other and then agreed on a consensus classification using four categories ("ACS and Transmural ischemia (TMI)", "ACS but not TMI", "Probably ACS", and "No signs of ACS"). The experts did not strictly apply conventional criteria such as ST-segment elevation of >0.1 mV in two adjacent limb leads or >0.2 mV in two adjacent precordial leads. Instead, they also considered the configuration of the ST-segment and the shape of the QRS complex, i.e. a pattern recognition analysis was applied as in the clinical routine interpretation of ECGs.

### Statistical analyses

The statistical analyses were conducted using SPSS release 12.0.1 (SPSS Inc, Chicago, U.S.). Odds ratios (OR) were calculated for the association between each potential risk factor and ACS. We regarded 95% confidence intervals (CIs) that excluded unity, or, equivalently, p < 0.05, as statistically significant. In the multivariable analysis, the probability of ACS was predicted using multiple logistic regression [27]. All independent variables except age were entered in the regression models as categorical variables, categorized as shown in tables 1, 2. In order to obtain meaningful baseline odds for ACS in the models, we chose 40 years old as reference value for age. Thus, we used the number of years above 40 as age variable in the models and allowed for both positive (patients above 40) and negative (patients below 40) numbers. Starting with the full multivariable model with all independent variables included, we excluded one insignificant independent variable at a time, starting with the variable with highest p-value, until only significant and important predictors remained. Categorical variables with more than two categories were kept in the model if the OR associated with any of the categories was significant. Variables with estimated OR of at least 2.5 (or, equivalently, at most 0.4) were considered as important predictors and thus kept in the model even if they were not statistically significant. The ECG variables were not considered for exclusion. For the final set of independent variables, categories with ORs close to one were collapsed with the reference category for that variable. Unknown response to one of the variables (hypertension) was also added to the reference category. We then assessed the significance of the interaction with age, implemented as cross-product terms together with main effects, for the following variables: sex, chest discomfort at presentation, symptom duration, and previous myocardial infarction. For comparison, we also established a prediction model where we replaced the ST amplitude characteristics of the ECG with the consensus ECG assessments by the two experts (LE and HÖ). Differences in specificity between the two models were tested using McNemar's test for correlated proportions.

The area under the receiver-operating-characteristic curve (ROC) was used as an overall measure of the discrimination abilities of the prediction models [28]. The area under ROC, measured in percent, can be interpreted as the probability that a randomly chosen patient with ACS has a higher outcome probability than a randomly chosen patient without ACS. For calculation of specificity and predictive values, the sensitivity was set to 95%. This somewhat arbitrary level was chosen because with current standard evaluation, some 2–5% of the ACS patients are erroneously discharged from the ED [29,30], which implies a sensitivity of at least 95 % for the routine ED work-up. In order to get more correct estimates of the generalization performance, i.e. the performance of the prediction models on an unseen study population, we used a *k*-fold cross-validation procedure [31]. Here we repeated times estimated the regression parameters based on randomly chosen training sets, comprising approximately 80% of all patients, and then evaluated the obtained model by calculating the area under ROC as well as the specificity at 95% sensitivity among the remaining 20% patients in a validation set kept completely isolated from the corresponding training set. This *k*-fold cross-validation procedure was implemented as follows: 1) the data set was randomly split in 5 groups of approximately equal size 2) the parameters of each model were estimated based on the patients of the training set only, which was established by excluding group *k *[*k *= 1, 2, ...., 5] 3) the group left out from parameter estimation was used as a validation set. We repeated the steps 1–3 above 20 times, which implied 20 * 5 = 100 sets of validations sets, 100 areas under ROC, and 100 estimates of the specificity at 95% sensitivity. Median values, together with 2.5 – 97.5 percentiles, from these 100 validation sets were used as measures of the generalization performance of the prediction models.

## Results

When each potential risk factor was considered separately, significant associations with ACS were observed for age, hypertension, previous myocardial infarction, chest discomfort at presentation, and symptom duration (table 1). Associations with ACS were also indicated for gender, angina pectoris, treatment with cardiac drugs, tachypnea, lung rales, and blood pressure, whereas no clear associations with smoking status, diabetes, congestive heart failure, previous PCI, previous CABG and heart rate were discerned. All ECG characteristics, ST amplitudes in leads I, aVF and V2, were markedly associated with ACS (table 2).

**Figure 1:**
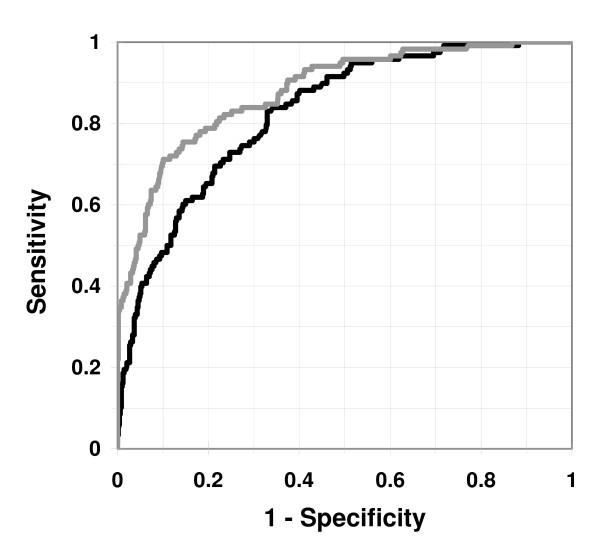
**Receiver-operating-characteristic curves (ROCs) for the prediction model with ECG and other clinical characteristics (black curve; area under ROC = 80.6%, n = 627^a^) and for the prediction model based on expert assessment of the ECG together with clinical characteristics (gray curve; area under ROC = 88.0%, n = 605^b^)**. ^a ^Data on at least one of the characteristics were missing for 7 (4 with ACS and 3 without ACS) of the original 634 patients. ^b ^Data on at least one of the clinical characteristics were missing for 3 (1 with and 2 without ACS) of the 608 patients with ECG assessed by the experts.

Many of the potential risk factors were strongly interrelated. In the multivariable analysis, older age, hypertension, angina pectoris, previous myocardial infarction, chest discomfort at presentation, short symptom duration, and abnormal ECG characteristics were associated with increased ACS risk (Table 3). Moreover, congestive heart failure and previous CABG were inversely associated with ACS. The prediction model based on these variables yielded a high discrimination percentage (area under ROC 80.6%; figure 1). For this model, hospitalizing all patients with a probability of ACS of at least 8.6%, corresponding to 65% of all patients, would yield a sensitivity of 95% and a specificity of 43%. This sensitivity-specificity pair would, with an ACS prevalence of 21% like in the present material, produce a negative predictive value (NPV) of 97% and a positive predictive value (PPV) of 30%. Adding the interaction terms related to age did not lead to any significant improvement of the model (results not shown).

**Table 3:** Multiple logistic regression model based on ECG and other clinical characteristics of patients (n = 627^a^) who come to the emergency department with acute chest pain and acute coronary syndrome (ACS). I-STamp, ST amplitude in lead I; aVF-STamp, ST amplitude in lead aVF; V2-STamp, ST amplitude in lead V2; STamp38, ST amplitude at the end of the third out of eight equal intervals between the ST-J point and the end of the T wave.

	Estimate	95% CI
Baseline odds for ACS^b^	0.0163	0.0073 – 0.0362
		
Odds ratios		
Age (no. of years above 40)	1.031	1.014 – 1.047
Hypertension	1.7	1.1 – 2.8
Angina pectoris ≤ 1 month	4.1	0.97 – 17
Congestive heart failure	0.48	0.24 – 0.94
Previous myocardial infarction		
Yes, ≤ 6 months	2.7	1.2 – 6.4
Yes, > 6 months	2.1	1.2 – 3.8
No	1.0	-
Previous CABG	0.23	0.09 – 0.60
Chest discomfort at presentation	1.9	1.2 – 3.1
Symptom duration		
0 – 6 h	3.8	2.0 – 7.1
7 – 12 h	2.8	1.2 – 6.5
> 12 h	1.0	-
I-Stamp		
I-STamp > 50 and I-STamp38 > I-Stamp	2.4	0.74 – 7.7
aVF-Stamp		
aVF-STamp>100 and aVF-STamp38>aVF-Stamp	9.4	2.7 – 33
aVF-STamp < -100 and aVF-STamp38 < aVF-STamp	4.1	0.72 – 23
None of above	1.0	
V2-Stamp		
V2-STamp > 200 and V2-STamp38 > V2-Stamp	3.4	1.5 – 7.4
100 < V2-STamp ≤ 200 and V2-STamp38 > V2-STamp	1.6	0.90 – 2.8
V2-STamp < -100 and V2-STamp38 < V2-Stamp	2.6	0.54 – 13
None of above	1.0	-

^a ^Data on at least one of characteristics were missing for 7 (4 with ACS and 3 without ACS) of the original 634 patients.

^b ^Baseline odds for ACS for a 40-year old patient who belongs to the reference category with respect to all other characteristics. The corresponding risk (probability) for ACS can be calculated as Odds/(1+Odds).

As an illustration of how the statistical model of table 3 can be used, consider patient A, a 72-year old female patient who seeks emergency care with ongoing chest discomfort that has lasted for 7 hours (table 4). She has undergone CABG previously and has had angina pectoris in connection with physical effort the last month. The ECG shows an elevation of the ST-amplitude above 200 in lead V2. The patient is 32 years above our chosen reference value for age (40 years old) and we therefore use 1.031^72–40 ^= 1.031^32 ^as age factor. Multiplying the ORs for the risk factors of this patient, the estimated odds for ACS can be obtained as 0.0163 × 1.031^32 ^× 4.1 × 0.23 × 1.9 × 2.8 × 3.4 ≈ 0.7385. Thus, the estimated probability of ACS for patient A is 0.7385/(1 + 0.7385) ≈ 0.42 = 42%. Patient B is a male 35-year old patient with chest discomfort for the last 72 hours and without any ST-elevations according to the ECG. This patient is 5 years below the reference value for age and we therefore use 1.031^35–40 ^= 1.031^-5 ^as age factor. The estimated odds for ACS for patient B is calculated as 0.0163 × 1.031^-5 ^× 1.9 ≈ 0.0266, which corresponds to a probability of ACS of 0.0266/(1 + 0.0266) ≈ 0.026 = 2.6% (table 4).

**Table 4:** Odds ratios for ACS for two hypothetical patients, A and B, used as examples. Patient A is female, 72 years old, and seeks emergency care with ongoing chest discomfort that has lasted for 7 hours. She has undergone CABG previously and has had angina pectoris in connection with physical effort the last month. The ECG shows an elevation of the ST-amplitude above 200 in lead V2 only. Patient B is male, 35-year old, and with chest discomfort for the last 72 hours but without any ST-elevations according to the ECG.

	Model Estimate	Patient A	OR	Patient B	OR
Baseline odds for ACS	0.0163		0.00163		0.00163
					
Odds ratios					
Age (no. of years above 40)	1.031	72	1.031^32^	35	1.031^-5^
Hypertension	1.7	No	1.0	No	1.0
Angina pectoris ≤ 1 month	4.1	Yes	4.1	No	1.0
Congestive heart failure	0.48	No	1.0	No	1.0
Previous myocardial infarction		No	1.0	No	1.0
Yes, ≤ 6 months	2.7				
Yes, > 6 months	2.1				
No	1.0				
Previous CABG	0.23	Yes	0.23	No	1.0
Chest discomfort at presentation	1.9	Yes	1.9	Yes	1.9
Symptom duration		7 hours	2.8	72 hours	1.0
0 – 6 h	3.8				
7 – 12 h	2.8				
> 12 h	1.0	-			
I-STamp		No	1.0	No	1.0
I-STamp > 50 and I-STamp38 > I-STamp	2.4				
aVF-STamp		No	1.0	No	1.0
aVF-STamp>100 and aVF-STamp38>aVF-STamp	9.4				
aVF-STamp < -100 and aVF-STamp38 < aVF-STamp	4.1				
None of above	1.0				
V2-STamp		> 200	3.4	No	1.0
V2-STamp > 200 and V2-STamp38 > V2-Stamp	3.4				
100 < V2-STamp ≤ 200 and V2-STamp38 > V2-STamp	1.6				
V2-STamp < -100 and V2-STamp38 < V2-STamp	2.6				
None of above	1.0	-			

### ECG assessments by experts

The ECG assessments made in consensus by the two experts were strong predictors of ACS (table 5), yielding an area under ROC of 76.1% with no other variables included in the prediction model. Adding other clinical data yielded markedly higher discriminating percentage (area under ROC 88.0%; table 6 and figure 2). For this model, a sensitivity of 95% would yield a specificity of 50%, which at an ACS-prevalence of 21% would produce a NPV of 98% and a PPV of 33%. The specificity in this model (50%) was significantly higher (p = 0.001) than in the model based on ECG amplitude data (43%). The discriminating percentage increased only marginally when the interaction terms related to age were incorporated (area under ROC 88.3%; regression model and ROC curve not shown).

**Table 5:** Expert assessment of the ECG for patients who come to the emergency department with acute chest pain, due to acute coronary syndrome (ACS; n = 120) or other causes (n = 488).

Assessment	ACS n (%)	Other causes n (%)	OR	95% CI
ACS and TMI^a^	30 (25.0)	3 (0.6)	94	28 – 320
ACS but not TMI^a^	10 (8.3)	9 (1.8)	10	4.0 – 27
Probably ACS	36 (30.0)	61 (12.5)	5.6	3.3 – 9.3
No signs of ACS	44 (36.7)	415 (85.0)	1.0	-

^a^TMI = Transmural ischemia

**Table 6:** Multiple logistic regression model based on expert assessment of the ECG together with clinical characteristics (n = 605)^a ^for the association between characteristics of ED chest pain patients and acute coronary syndrome (ACS).

	Estimate	95% CI
Baseline odds for ACS^b^	0.0066	0.0024 – 0.0178
		
Odds ratios		
Age (no. of years above 40)	1.036	1.016 – 1.057
Hypertension	2.3	1.3 – 4.1
Angina pectoris ≤ 1 month	2.8	0.58 – 14
Congestive heart failure	0.55	0.26 – 1.2
Previous myocardial infarction		
Yes, ≤ 6 months	3.4	1.3 – 8.7
Yes, > 6 months	1.9	0.99 – 3.7
No	1.0	-
Previous CABG	0.28	0.10 – 0.75
Chest discomfort at presentation	1.8	1.0 – 3.1
Symptom duration		
0 – 6 h	4.6	2.2 – 9.6
7 – 12 h	3.7	1.4 – 10
> 12 h	1.0	-
ECG expert assessment		
ACS and TMI	97	26 – 360
ACS but not TMI	11	3.5 – 37
Probably ACS	5.8	3.2 – 11
No signs of ACS	1.0	-

^a ^Data on at least one of characteristics were missing for 3 (1 with and 2 without ACS) of the 608 patients with ECG assessed by the experts.

^b ^Baseline odds for ACS for a 40-year old patient who belongs to the reference category with respect to all other characteristics. The corresponding risk (probability) for ACS can be calculated as Odds/(1+Odds).

### Cross-validation

Cross-validating the model including clinical and ECG amplitude data decreased the area under ROC from 80.6% to 76.8% in median (2.5 – 97.5 percentiles 69.1 – 84.1%), and lowered the specificity from 43% to 35% in median (2.5 – 97.5 percentiles 13 – 60%) if the sensitivity was set to 95%. This median specificity at 95% sensitivity would, with a prevalence of ACS of 21%, diminish the NPV from 97% to 96% (2.5 – 97.5 percentiles 91 – 98%) and the PPV from 30% to 27% (2.5 – 97.5 percentiles 22 – 38%). Cross-validating the corresponding model based on expert ECG assessments lowered the area under ROC from 88.0% to 85.7% in median (2.5 – 97.5 percentiles 78.5 – 93.0%). By contrast, the specificity at 95% sensitivity was unchanged (50% in median, 2.5 – 97.5 percentiles 21 – 74%), yielding the same NPV (98% in median; 2.5 – 97.5 percentiles 94 – 98%) and the same PPV (33% in median; 2.5 – 97.5 percentiles 24 – 49%) as before.

## Discussion

The present study was undertaken to develop a simple and user-friendly statistical model for ACS prediction in the ED. The model was developed using logistic regression on clinical and ECG data from chest pain patients at the ED of a university hospital. Simple categorized ECG amplitude data were included, which allows automated ECG reading, makes ACS prediction stable, and makes the model useful for personnel inexperienced in ECG reading. To keep the model simple and immediately usable in the ED, results from blood samples were not included. Besides ECG, the following 8 variables were found to be important for ACS prediction, and included in the model: age, chest discomfort at presentation, symptom duration and previous hypertension, angina pectoris, AMI, congestive heart failure or PCI/CABG. The interaction terms, which involved only clinical characteristics and not the ECG amplitude data, did not improve the performance substantially and were therefore not included in the final model.

Many of the variables included in the present model have previously appeared in models for AMI or ACS prediction. All previous models have included the ECG, and there is a general consensus that the ECG is the most important clinical variable to predict ACS in the ED [32,33]. Most models have also had a factor for previous coronary artery disease, e.g[13,15,18-21], many have included age[12,14,18,19,23] and pain duration [12,14,18,23], but few have included congestive heart failure [19] or hypertension [18]. Other variables that have appeared in previous models are diabetes [12,18,19], sex [14,18], pain localization [12,14,15,18,20,22] pain similar to previous angina or AMI [15,18], the quality of the pain [12,15,18], and previous use of nitrates [18,20]. A systematic review of studies on AMI probability [34] has shown that additional variables with predictive power may be a third heart sound, hypotension, chest pain reproduced by palpation and positional chest pain.

With current standard evaluation in the ED, some 2–5% of the ACS patients are erroneously discharged from the ED [29,30], which indicates a sensitivity of at least 95 % for the routine ED work-up. Set to this level of sensitivity, our simple model reached a specificity of about 35%, and a PPV and NPV of 96% and 27%, respectively, in the cross-validation. In the present patient material, a sensitivity of 95% could be reached by admitting all patients with an ACS probability above 8.6%, which would not imply a significant change of the actual admission rate. A PPV of 27% may seem very low, but it is likely at a level similar to that of the ED physician's decision after the current standard ED assessment, where some 70% of those admitted for suspected ACS prove not to have it [1,2,35]. We have been unable to find any published data on the PPV of standard ED assessment for possible ACS. Thus, our model did not seem to perform any better than the average physician, and will therefore likely not be useful for the expert physician in the ED. However, if a simple prediction model with automated ECG reading, like ours, can maintain a NPV of 96% in a larger prospective study, we believe it can be useful in real-life routine care. In addition, in healthcare settings with an ACS prevalence below 21% among chest pain patients [2,36], NPV may increase. Seemingly better performances have been reported for previous ACS prediction models [8,12,14,24], but some of these models were both developed and tested in chest pain populations with clearly higher prevalence of ACS than ours. Further, with the exception of the ACI-TIPI [14], these models required the input of a large number of variables, and also ECG interpretation by the ED physician.

The model in the present study with ECG interpretation by experts had much higher overall discrimination ability (area under ROC) and specificity at 95% sensitivity, whereas its predictive ability was only somewhat better than the model based on ST amplitude data, with PPV and NPV of 33 vs 27% and 96 vs 98%. The significance of these differences, translated into a real-world clinical practice, is probably small. It is worth noting that basing a prediction model only on the categorized ST amplitude data would not yield sufficient predictive power since about half (52%; not in results) of the ACS patients were in fact normal according to the used cut-offs for the ST amplitudes in all three leads (I, aVF and V2). However, the combination of simple categorized ST amplitude data and clinical characteristics resulted in almost as much predictive power as expert interpretation of the entire ECG. Further, since the average ED physician is less experienced in ECG interpretation than our experts, the model with computerized ECG reading is probably a better choice for clinical implementation. ACS prediction will be more stable, and personnel with little or no experience in ECG reading, e.g. triage nurses, can use the model with no loss in predictive power. Incorporating ST amplitude data as numeric measurements rather than as categorizations and then allowing for interaction between the ECG measures would most likely increase the predictive power of the statistical model further. In addition, since we did not test all theoretically possible ECG measurements, it may be that additional ECG variables or combinations of variables could have increased the predictive power of the ECG and the entire model further. An important disadvantage of a more complex prediction model is however that the rationale behind its output will be much more difficult to understand by potential users.

A model for ACS prediction based on automated ECG reading together with clinical characteristics can probably be applied in many different settings. For the present model, it seems wise to exploit the NPV, since PPV was low at a sensitivity of 95%. If our model can be validated in prospective studies, it is thus probably best used as support for discharging patients in settings where the ACS prevalence is low, e.g. in primary care, in the initial ED triage or in telemedicine situations, where information is limited. It would also seem possible to use the model as support for the early discharge of low risk patients after a brief observation in the ED or in a chest pain unit, or to assist inexperienced junior staff in the ED, as a means to improve quality of care. Adding results of blood samples, repeated ECGs and physician judgment would probably increase NPV to close to 100%. Whatever the use of our model, a good feature is the limited number of variables, which implies a small need for manual input, and an increased likelihood that the model will actually be used in a busy healthcare environment. With the exception of the ACI-TIPI model [14], the need for a time-consuming large input has been a weak point of several earlier prediction models [8-12,18], where up to 40 questions need to be answered before the model gives decision support. Our model needs answers to 8 questions.

### Limitations of the study

The patients included in the present model were retrospectively collected and from one center only. Because of the retrospective nature of the study, there may of course be errors despite a careful collection of all data. For example, the diagnoses of the patients discharged from the ED were not tested with routine post-discharge ECG or blood samples for cardiac markers. Therefore, before clinical implementation, the model clearly needs to be validated prospectively, preferably at multiple centers. An old definition of AMI was used in the present study. Newer definitions of AMI have lower cut-off values for biochemical markers, and some of the unstable angina diagnoses in this study would currently be classified as AMI [37]. However, the total number of ACS cases would probably be little changed. Furthermore, the baseline risk (odds) for ACS of the model has to be tuned in each population before implementation. Since it is impossible to analyze all information with a potential impact on the likelihood of ACS, it is of course possible that variables in addition to those included in our model could be important for ACS prediction. The size of the sample might also have limited our abilities to detect clinical or ECG characteristics of low prevalence that nevertheless are important for ACS classification.

## Conclusion

Previous studies have shown that decision support systems are an effective means of improving patient care [38]. The aim of this study was to create a simple and practical statistical model for ACS prediction that could be used in routine care at a busy ED. The model includes simple ECG data (suitable for computerized reading) and 8 clinical variables and can be used immediately on patient arrival by personnel inexperienced in ECG interpretation. At the set sensitivity of 95% and an ACS prevalence of 21%, the NPV was 96%. We believe that this prediction model, combined with the judgment of trained healthcare personnel, could be useful for the early discharge of chest pain patients in populations with a low prevalence of ACS. However, the model must be prospectively validated before it can be used in clinical practice.

## Competing interests

The author(s) declare that they have no competing interests.

## Authors' contributions

All authors participated in the design of the study. JB performed the statistical analyses and drafted the manuscript. JLF was involved in data acquisition and assisted in drafting the manuscript. MO assisted in the statistical analyses. LE and HÖ performed the ECG assessments. UE coordinated the study team, was involved in data acquisition, and drafted the manuscript. All authors read the manuscript critically and approved the final version.

## Pre-publication history

The pre-publication history for this paper can be accessed here:



## References

[B1] Pope JH, Ruthazer R, Beshansky JR, Griffith JL, Selker HP (1998). Clinical Features of Emergency Department Patients Presenting with Symptoms Suggestive of Acute Cardiac Ischemia: A Multicenter Study. J Thromb Thrombolysis.

[B2] Ekelund U, Nilsson HJ, Frigyesi A, Torffvit O (2002). Patients with suspected acute coronary syndrome in a university hospital emergency department: an observational study. BMC Emerg Med.

[B3] Forberg JL, Henriksen LS, Edenbrandt L, Ekelund U (2006). Direct hospital costs of chest pain patients attending the emergency department: a retrospective study. BMC Emerg Med.

[B4] Kontos MC, Schmidt KL, McCue M, Rossiter LF, Jurgensen M, Nicholson CS, Jesse RL, Ornato JP, Tatum JL (2003). A comprehensive strategy for the evaluation and triage of the chest pain patient: a cost comparison study. J Nucl Cardiol.

[B5] Chandra A, Rudraiah L, Zalenski RJ (2001). Stress testing for risk stratification of patients with low to moderate probability of acute cardiac ischemia. Emerg Med Clin North Am.

[B6] Udelson JE, Beshansky JR, Ballin DS, Feldman JA, Griffith JL, Handler J, Heller GV, Hendel RC, Pope JH, Ruthazer R, Spiegler EJ, Woolard RH, Selker HP (2002). Myocardial perfusion imaging for evaluation and triage of patients with suspected acute cardiac ischemia: a randomized controlled trial. JAMA.

[B7] Kontos MC (1999). Role of Echocardiography in the Emergency Department for Identifying Patients with Myocardial Infarction and Ischemia. Echocardiography.

[B8] Baxt WG, Shofer FS, Sites FD, Hollander JE (2002). A neural network aid for the early diagnosis of cardiac ischemia in patients presenting to the emergency department with chest pain. Ann Emerg Med.

[B9] Baxt WG, Shofer FS, Sites FD, Hollander JE (2002). A neural computational aid to the diagnosis of acute myocardial infarction. Ann Emerg Med.

[B10] Kennedy RL, Burton AM, Fraser HS, McStay LN, Harrison RF (1996). Early diagnosis of acute myocardial infarction using clinical and electrocardiographic data at presentation: Derivation and evaluation of logistic regression models. Eur Heart J.

[B11] Kennedy RL, Harrison RF, Burton AM, Fraser HS, Hamer WG, MacArthur D, McAllum R, Steedman DJ (1997). An artificial neural network system for diagnosis of acute myocardial infarction (AMI) in the accident and emergency department: Evaluation and comparison with serum myoglobin measurements. Comput Methods Programs Biomed.

[B12] Kennedy RL, Harrison RF (2005). Identification of patients with evolving coronary syndromes using statistical models with data from the time of presentation. Heart.

[B13] Tierney WM, Roth BJ, Psaty B, McHenry R, Fitzgerald J, Stump DL, Anderson FK, Ryder KW, McDonald CJ, Smith DM (1985). Predictors of myocardial infarction in emergency room patients. Crit Care Med.

[B14] Selker HP, Beshansky JR, Griffith JL, Aufderheide TP, Ballin DS, Bernard SA, Crespo SG, Feldman JA, Fish SS, Gibler WB, Kiez DA, McNutt RA, Moulton AW, Ornato JP, Podrid PJ, Pope JH, Salem DN, Sayre MR, Woolard RH (1998). Use of the acute cardiac ischemia time-insensitive predictive instrument (ACI-TIPI) to assist with triage of patients with chest pain or other symptoms suggestive of acute cardiac ischemia. A multicenter, controlled clinical trial. Ann Intern Med.

[B15] Goldman L, Cook EF, Brand DA, Lee TH, Rouan GW, Weisberg MC, Acampora D, Stasiulewicz C, Walshon J, Terranova G (1988). A computer protocol to predict myocardial infarction in emergency department patients with chest pain. N Engl J Med.

[B16] Goldman L, Cook EF, Johnson PA, Brand DA, Rouan GW, Lee TH (1996). Prediction of the need for intensive care in patients who come to emergency departments with acute chest pain. N Engl J Med.

[B17] Karlson BW, Herlitz J, Hallgren P, Liljeqvist JA, Oden A, Hjalmarson A (1994). Emergency room prediction of mortality and severe complications in patients with suspected acute myocardial infarction. Eur Heart J.

[B18] Aase O, Jonsbu J, Liestol K, Rollag A, Erikssen J (1993). Decision support by computer analysis of selected case history variables in the emergency room among patients with acute chest pain. Eur Heart J.

[B19] Lopez de Sa E, Lopez-Sendon J, Anguera I, Bethencourt A, Bosch X (2002). Prognostic value of clinical variables at presentation in patients with non-ST-segment elevation acute coronary syndromes: results of the Proyecto de Estudio del Pronostico de la Angina (PEPA). Medicine (Baltimore).

[B20] Pozen MW, D'Agostino RB, Selker HP, Sytkowski PA, Hood WBJ (1984). A predictive instrument to improve coronary-care-unit admission practices in acute ischemic heart disease. A prospective multicenter clinical trial. N Engl J Med.

[B21] Grijseels EWM, Deckers JW, Hoes AW, Hartman JAM, Vanderdoes E, Vanloenen E, Simoons ML (1995). Prehospital Triage of Patients with Suspected Myocardial- Infarction - Evaluation of Previously Developed Algorithms and New Proposals. Eur Heart J.

[B22] Grijseels EWM, Deckers JW, Hoes AW, Boersma E, Hartman JAM, vanderDoes E, Simoons ML (1996). Implementation of a pre-hospital decision rule in general practice - Triage of patients with suspected myocardial infarction. Eur Heart J.

[B23] Mair J, Smidt J, Lechleitner P, Dienstl F, Puschendorf B (1995). A decision tree for the early diagnosis of acute myocardial infarction in nontraumatic chest pain patients at hospital admission. Chest.

[B24] Harrison RF, Kennedy RL (2005). Artificial neural network models for prediction of acute coronary syndromes using clinical data from the time of presentation. Ann Emerg Med.

[B25] Tunstall-Pedoe H, Kuulasmaa K, Amouyel P, Arveiler D, Rajakangas AM, Pajak A (1994). Myocardial infarction and coronary deaths in the World Health Organization MONICA Project. Registration procedures, event rates, and case-fatality rates in 38 populations from 21 countries in four continents. Circulation.

[B26] Van de Werf F, Ardissino D, Betriu A, Cokkinos DV, Falk E, Fox KA, Julian D, Lengyel M, Neumann FJ, Ruzyllo W, Thygesen C, Underwood SR, Vahanian A, Verheugt FW, Wijns W (2003). Management of acute myocardial infarction in patients presenting with ST-segment elevation. The Task Force on the Management of Acute Myocardial Infarction of the European Society of Cardiology. Eur Heart J.

[B27] Hosmer DW, Lemeshow S (2000). Applied logistic regression.

[B28] Hanley JA, McNeil BJ (1982). The meaning and use of the area under a receiver operating characteristic (ROC) curve. Radiology.

[B29] Lee TH, Rouan GW, Weisberg MC, Brand DA, Acampora D, Stasiulewicz C, Walshon J, Terranova G, Gottlieb L, Goldstein-Wayne B (1987). Clinical characteristics and natural history of patients with acute myocardial infarction sent home from the emergency room. Am J Cardiol.

[B30] Pope JH, Aufderheide TP, Ruthazer R, Woolard RH, Feldman JA, Beshansky JR, Griffith JL, Selker HP (2000). Missed diagnoses of acute cardiac ischemia in the emergency department. N Engl J Med.

[B31] Baumann K (2003). Cross-validation as the objective function for variable-selection techniques. TRAC-Trends in Analytical Chemistry.

[B32] Goldman L, Kirtane AJ (2003). Triage of patients with acute chest pain and possible cardiac ischemia: the elusive search for diagnostic perfection. Ann Intern Med.

[B33] Lee TH, Goldman L (2000). Evaluation of the patient with acute chest pain. N Engl J Med.

[B34] Panju AA, Hemmelgarn BR, Guyatt GH, Simel DL (1998). The rational clinical examination. Is this patient having a myocardial infarction?. JAMA.

[B35] Karlson BW, Herlitz J, Wiklund O, Richter A, Hjalmarson A (1991). Early prediction of acute myocardial infarction from clinical history, examination and electrocardiogram in the emergency room. Am J Cardiol.

[B36] Karlson BW, Karlsson E Bröstsmärta. State of the Art.

[B37] Trevelyan J, Needham EW, Smith SC, Mattu RK (2004). Impact of the recommendations for the redefinition of myocardial infarction on diagnosis and prognosis in an unselected United Kingdom cohort with suspected cardiac chest pain. Am J Cardiol.

[B38] Kawamoto K, Houlihan CA, Balas EA, Lobach DF (2005). Improving clinical practice using clinical decision support systems: a systematic review of trials to identify features critical to success. BMJ.

